# Learning Curve of Ultrasound-Guided Percutaneous Needle Biopsy for Pleural Lesions: A Retrospective Study at Two Tertiary Referral Hospitals

**DOI:** 10.3390/diagnostics15131613

**Published:** 2025-06-25

**Authors:** Byunggeon Park, Jihoon Hong, Seo Young Park, See Hyung Kim, Jae-Kwang Lim, An Na Seo, Seung-Ick Cha, Jaehee Lee, Ji Eun Park, Haewon Jung, Jongmin Park

**Affiliations:** 1 Department of Radiology, School of Medicine, Kyungpook National University, Daegu 41944, Republic of Korea; redzon7543@gmail.com (B.P.);; 2 Department of Pathology, School of Medicine, Kyungpook National University, Daegu 41944, Republic of Korea; 3 Department of Internal Medicine, School of Medicine, Kyungpook National University, Daegu 41944, Republic of Korea; 4 Department of Emergency Medicine, School of Medicine, Kyungpook National University, Daegu 41944, Republic of Korea

**Keywords:** pleura, ultrasound-guided biopsy, learning curve

## Abstract

**Background/Objectives**: Ultrasound (US)-guided percutaneous pleural needle biopsy (PCPNB) is increasingly performed as a minimally invasive approach for the diagnosis of pleural lesions. However, no prior studies have investigated the learning curve for this method. The purpose of this study was to assess the learning curve of US-guided PCPNB using the cumulative summation (CUSUM) method and to calculate the number of procedures to achieve proficiency. **Methods**: A retrospective analysis was performed on 491 US-guided PCPNBs performed by four board-certified radiologists at two tertiary referral hospitals from January 2012 to December 2024. The standard and risk-adjusted CUSUM (RA-CUSUM) techniques were used to evaluate diagnostic success and false-negative rates. The potential impact of previous percutaneous needle biopsy (PCNB) experience on the learning curve was also assessed. **Results**: The overall diagnostic success and false-negative rate were 89.2% (438/491) and 8.1% (40/491), respectively. Operators achieved acceptable diagnostic success in US-guided PCPNB after a median of 15 (range, 12–45) procedures in standard CUSUM analysis and 22 (range, 10–33) procedures in RA-CUSUM analysis. Acceptable false-negative rates were attained after 18 (range, 14–42) and 24 (range, 12–44) procedures, respectively. Operators with prior PCNB experience required 12 procedures to achieve both acceptable diagnostic success and an acceptable false-negative rate. In contrast, those without experience required 29 and 27 procedures, respectively. Complications occurred in 1.4% (7/491), including one major complication (0.2%). **Conclusions**: Diagnostic proficiency and procedural safety in performing US-guided PCPNB improved with increasing operator experience. The low complication rate highlights the clinical safety and feasibility of US-guided PCPNB.

## 1. Introduction

The annual global incidence of pleural disease is estimated at 3.5–10 million [1]. Pleural lesions primarily originate from metastatic disease of pulmonary or extrapulmonary origin, tuberculous pleurisy, and parapneumonic effusions [2]. For indeterminate pleural abnormalities, pleural fluid analysis is useful in distinguishing transudative from exudative effusions, aiding treatment decisions [3]. However, this method has limitations, and while thoracoscopy and video-assisted thoracic surgery offer definitive diagnoses, they are costly, less accessible, and associated with complications [4]. As a less invasive alternative, image-guided percutaneous needle biopsy (PCNB) for pleural lesions has been widely performed [5,6,7].

Image-guided percutaneous pleural needle biopsy (PCPNB) is a widely accepted diagnostic method for evaluating pleural lesions, with both CT and ultrasound (US) commonly used as guidance modalities [6,7]. CT-guided PCPNB allows for accurate preprocedural planning of the needle path using high-resolution static imaging. However, it lacks real-time feedback during the procedure, making it difficult to respond to respiratory motion or adjust needle direction dynamically [8]. In contrast, US-guided PCPNB enables continuous real-time visualization of both the pleura and the biopsy needle. This facilitates dynamic adjustment during respiration and eliminates radiation exposure, which may contribute to improved targeting and a lower complication rate in appropriately selected patients [6,9]. Importantly, the procedural nature of US-guided PCPNB differs from that of CT-guided techniques—not only in imaging modality but also in the demands placed on the operator during the procedure. Real-time US guidance requires simultaneous image interpretation and needle manipulation, which may influence how operators achieve and maintain consistent diagnostic performance and procedural safety. While learning curves for CT-guided PCNB have been investigated in previous studies, no such data exist for US-guided PCPNB [10,11]. Given the increasing use of US guidance in clinical practice and its distinct procedural characteristics, an objective evaluation of the learning curve for US-guided PCPNB is warranted.

The cumulative summation (CUSUM) method has been widely utilized as a statistical tool for assessing learning curves and monitoring procedural proficiency [12]. This methodology has been increasingly adopted in interventional radiology, particularly for evaluating procedural competence during image-guided interventions. Learning curve studies for US-guided biopsies of other organs, including the liver, kidney, and lung, have shown that procedural proficiency is typically achieved after a variable number of cases, depending on organ-specific and lesion-related factors [13,14,15]. While such findings provide useful context, the learning curve for US-guided PCPNB has not been systematically evaluated. Although several studies have applied CUSUM analysis to CT-guided thoracic procedures, its application to US-guided PCPNB remains unexplored [10,11]. There is a clear lack of data on this topic, despite the growing clinical use of US guidance for pleural biopsy. Addressing this limitation is important for establishing appropriate training protocols and ensuring consistent diagnostic performance and procedural safety in practice.

## 2. Materials and Methods

This retrospective study was approved by the Institutional Review Board of Kyungpook National University Hospital and Kyungpook National University Chilgok Hospital, which waived the requirement for informed patient consent.

### 2.1. Patients

From January 2012 to December 2024, 506 US-guided PCPNBs were performed by four board-certified radiologists at Kyungpook National University Hospital and Kyungpook National University Chilgok Hospital, Daegu, South Korea. All patients’ medical records, procedural records, US findings during PCPNB, post-PCPNB follow-up studies, and diagnoses of biopsied lesions were retrospectively reviewed in consensus by two thoracic radiologists (J.P. and B.P., each with 11 years of experience).

### 2.2. Biopsy Procedure

To ensure proper sterilization, the US transducer was disinfected with 10% iodopovidone for a minimum of 2 min before the procedure. The patient’s position was determined based on the lesion’s location to allow the most effective access. US-guided PCPNBs were conducted using two different US systems (iU22 and EPIQ Elite; Philips Healthcare, Bothell, WA, USA). Transducer selection was adjusted according to the patient’s body habitus and the lesion’s visibility, using either a low-frequency (1–5 MHz) or high-frequency (10–12 MHz) probe. “Prior to each procedure, ultrasound examination was performed during both inspiration and expiration, and the respiratory phase that provided optimal lesion visualization was selected for biopsy”.

Real-time color Doppler imaging was employed to minimize the risk of vascular injury. After administering local anesthesia, the biopsy needle was inserted and guided toward the lesion using a freehand approach. The number of acquisitions was determined based on specimen quality and patient tolerance. An 18-gauge automated core biopsy needle (Acecut; TSK Laboratory, Tochigi, Japan), which is not specifically designed as an echogenic needle, was utilized without a coaxial system, with a throw length of 1.1 cm selected at the radiologist’s discretion. All collected specimens were immediately preserved in 10% formalin and submitted for histopathological evaluation.

### 2.3. Outcome Assessment

The biopsied lesion was classified as nondiagnostic, benign, or malignant. Nondiagnostic lesions included cases where the obtained tissue was insufficient for diagnosis, such as fibromuscular tissue or skeletal muscle. The final diagnosis of each biopsied lesion was determined based on biopsy specimens, surgical pathology, or follow-up imaging: (1) For patients who underwent surgical resection, the pathological findings from the surgical specimen were used as the final diagnosis. (2) Lesions diagnosed as malignant or as specific benign entities (e.g., tuberculosis or hamartoma) based on biopsy pathology were accepted as the final diagnosis. (3) Lesions with nonspecific benign pathology were classified as benign if they demonstrated a ≥20% reduction in diameter during follow-up or remained stable for at least two years. (4) Lesions with nonspecific benign pathology that did not meet these criteria or had a follow-up period of less than two years were considered indeterminate. All specimens were reviewed by board-certified pathologists, with immunohistochemistry or molecular studies performed when necessary to confirm or further characterize malignancy. Lesions classified as benign or malignant were categorized into true positive, true negative, false positive, or false negative based on US-guided PCPNB results.

Immediately after the procedure, US was performed to assess acute complications, including bleeding, pneumothorax, and hematoma. A chest radiograph was taken at 3 and 24 h post-biopsy to detect pneumothorax. Post-procedural complications were assessed in the hospitalization period and recorded as minor (class A–B) or major (class C–F) [16].

### 2.4. Statistical Analysis

The standard and risk-adjusted cumulative sum (CUSUM and RA-CUSUM) methods were used to assess the learning curve for US-guided PCPNBs in terms of diagnostic performance [17]. For the diagnostic accuracy curve, accurate diagnoses (true positives and true negatives) were classified as ‘success’. In contrast, cases with a nondiagnostic specimen or those classified as false-positive or false-negative for malignancy were considered ‘failure’. Indeterminate cases were excluded from the analysis. For the false-negative rate curve, benign lesions were excluded; therefore, successes included only true-positive cases, while failures included only nondiagnostic specimens and false negatives. The standard CUSUM was constructed to classify cases as success or failure based on the occurrence of complications.

The CUSUM score(s) was calculated based on p0, representing an acceptable failure rate (reflecting inherent error when the procedure is performed correctly), and p1, representing an unacceptable failure rate. The initial CUSUM score was set to zero. With each successful case, the score decreased by s, whereas with each failure, the score increased by 1-s. Learning was considered achieved when the CUSUM line crossed the lower decision threshold (H0). If the line exceeded the upper decision threshold (H1), it indicated a failure to learn. When the CUSUM line remained between H0 and H1, learning was deemed inconclusive.

For both standard CUSUM and RA-CUSUM analyses, the acceptable failure rate thresholds were initially set at p_0_ = 0.16 and p_1_ = 0.32 (i.e., double the acceptable failure rate) based on the prior literature [6,17]. To assess the learning curve under stricter criteria, additional analyses were conducted using a more stringent failure rate of p_0_ = 0.12 and p_1_ = 0.24. The acceptable complication occurrence rate was defined as 0.02, whereas the unacceptable complication occurrence rate was set at 0.06 [6,18]. The risk for type I and type II errors was set at 0.1 for all analyses.

RA-CUSUM analysis was performed to account for the varying difficulty levels of each procedure. In RA-CUSUM, the score (s_i_) was determined based on the predicted failure risk (p_i_) for each procedure, calculated using a logistic regression model incorporating baseline variables known to influence diagnostic performance. Variables including sex, age, pleural lesion morphology (nodular vs. diffuse), pleural thickness (<4.5 mm vs. ≥4.5 mm), and number of biopsy cores obtained were initially selected based on a univariable analysis with *p* < 0.20 and then entered into a multivariable logistic regression model. Among these, variables with statistical significance (*p* < 0.05) were used for RA-CUSUM adjustment [7,19]. Operators were stratified into subgroups based on prior US-guided PCNB experience during residency. Prior experience included freehand US-guided biopsies of various organ systems, including the liver, lung, and kidney, whereas the inexperienced group had no such procedural experience during residency (Table S1). The CUSUM curve was calculated by averaging the individual operators’ CUSUM values within each subgroup, including only data up to the minimum procedure count among operators in each group, with values beyond this threshold excluded.

The CUSUM curves were constructed using Microsoft Excel 2016 (Microsoft Corporation, Redmond, WA, USA). All statistical analyses were performed using the Statistical Package for the Social Sciences (SPSS version 23.0, IBM Corp., Armonk, NY, USA). For all statistical analyses, the level of significance was set at values of *p* < 0.05.

## 3. Results

### 3.1. Patient and Lesion Characteristics

**Figure 1:**
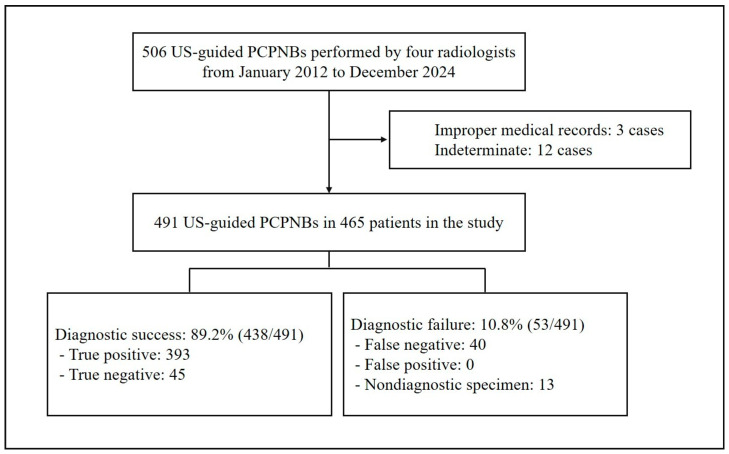
Flowchart of the study. PCPNB = percutaneous pleural needle biopsy.

Of the 506 US-guided PCPNBs, 15 cases were excluded: 12 due to indeterminate diagnosis (including 7 patients lost to follow-up and 5 with follow-up periods shorter than two years) and 3 due to improper medical records, defined as incomplete documentation of pathology results (Figure 1). The four operators performed a total of 491 US-guided PCPNBs in 465 patients during the study period (Table 1). The mean patient age was 70.3 ± 12.1 (SD) years (median, 72 years; range, 23–98 years). The sample included 300 (64.5%) men and 165 (35.5%) women. Among the 465 patients, 20 underwent two US-guided PCPNBs, and three underwent three US-guided PCPNBs. The mean pleural thickness was 9.6 ± 6.6 mm (median, 7.9 mm; range, 1.3–47.2 mm); 94 (19.1%) lesions were < 4.5 mm in thickness. The pleural morphology was classified as nodular in 343 cases (69.9%) and diffuse in 148 cases (30.1%).

### 3.2. Learning Curves for Diagnostic Performance

The 491 US-guided PCPNBs were classified as malignant (90.8%, 446/491) or benign (9.2%, 45/491) based on the available pathology reporting and imaging follow-up. Among benign lesions with more than two years of imaging follow-up, the median follow-up duration was 42 months (range, 27–101). The most common malignant diagnoses were lung cancer (83.4%, 372/446) and metastasis (8.5%, 38/446). The most common benign diagnosis was tuberculosis (53.3%, 24/45). The 491 US-guided PCPNBs included 53 diagnostic failures (10.8%): 0 false positives, 40 false negatives, and 13 nondiagnostic specimens. Among the 40 false-negative cases, the final diagnosis was established by repeat pleural biopsy in 18 cases (45%), repeat biopsy from another site in 21 cases (52.5%), and surgical confirmation of a lung lesion in 1 case (2.5%). The remaining 438 cases (89.2%) were correctly diagnosed and classified as cases of diagnostic success, of which 393 were true positives and 45 were true negatives. The overall diagnostic success rates and false negative rates are described in Table 2.

**Figure 2:**
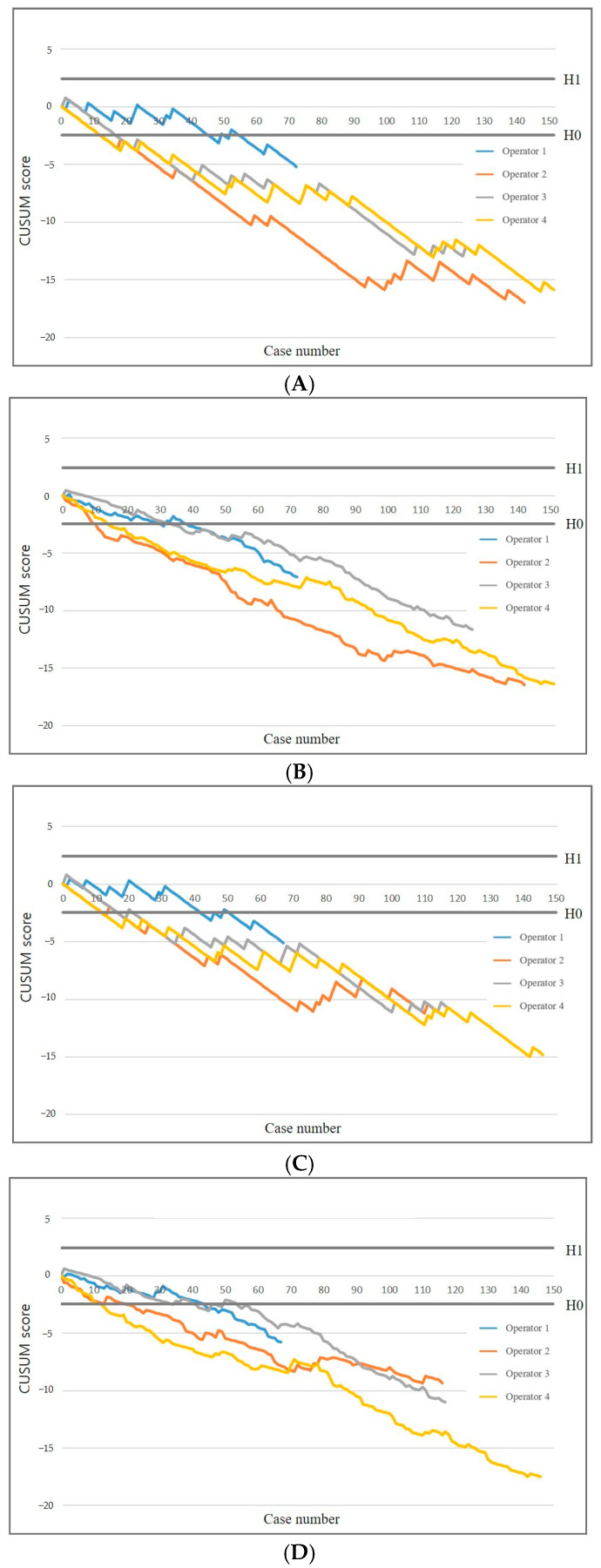
CUSUM graphs of diagnostic success and false negative rate. Standard (**A**) and risk-adjusted (**B**) CUSUM graphs of diagnostic success. Standard (**C**) and risk-adjusted (**D**) CUSUM graphs of false negative rate. Diagnostic success results in a downward trend, whereas diagnostic failures lead to an upward trend. CUSUM = cumulative summation, H0 = lower decision limit, H1 = upper decision limit.

Figure 2 presents the standard and RA-CUSUM charts illustrating the diagnostic success of each operator. Multivariable logistic regression identified nodular morphology and pleural thickness ≥4.5 mm as independent predictors of diagnostic success in US-guided PCPNB (*p* < 0.05). All operators achieved an acceptable failure rate of 16% in diagnostic success after a median number of 15 US-guided PCPNB procedures (range, 12–45) (Table 3). The RA-CUSUM plots showed a pattern similar to that of the standard CUSUM plots. All operators attained an acceptable failure rate after a median of 22 US-guided PCPNB procedures (range, 10–33). All operators achieved an acceptable false-negative rate, requiring a median of 18 procedures (range, 14–42) in the standard CUSUM analysis. In the RA-CUSUM analysis, the median number of procedures required was 24 (range, 12–44). Diagnostic accuracy continued to improve as operators performed additional procedures beyond the threshold for acceptable accuracy.

**Figure 3:**
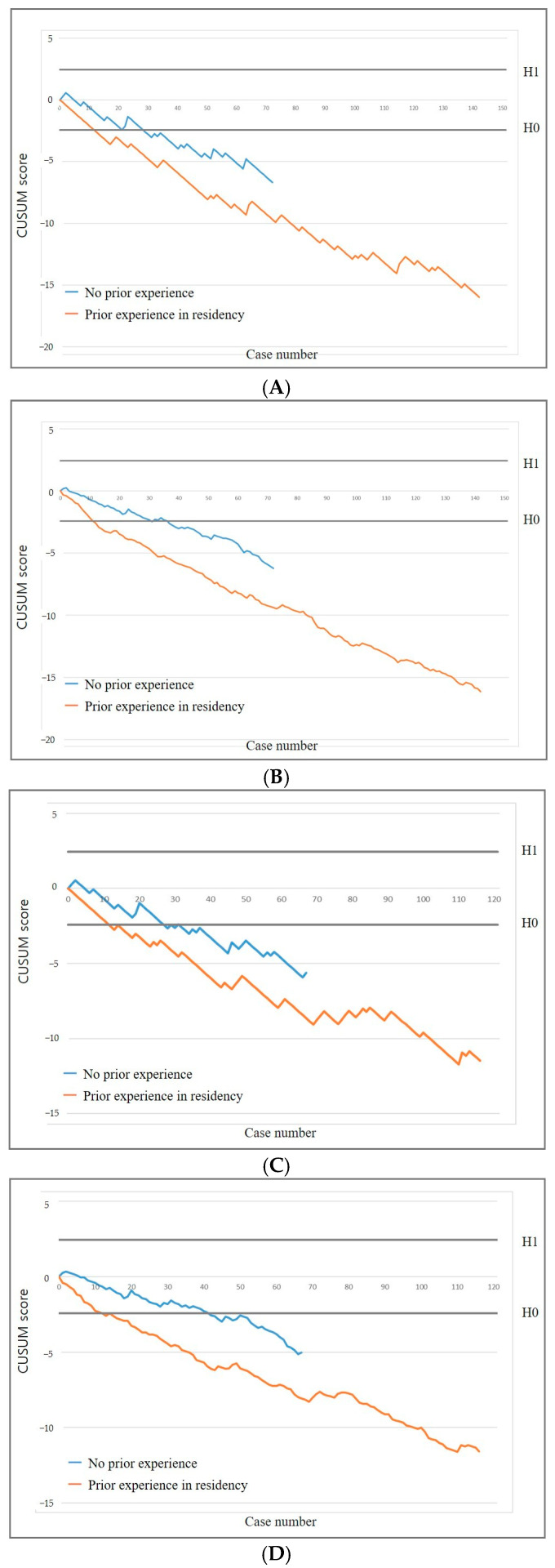
CUSUM graphs stratified by operator subgroups based on prior experience in percutaneous needle biopsy during radiology residency. Standard (**A**) and risk-adjusted (**B**) CUSUM graphs of diagnostic success. Standard (**C**) and risk-adjusted (**D**) CUSUM graphs of false negative rate.

Operators with prior US-guided PCNB experience required 12 procedures, while those without required 29 to achieve an acceptable diagnostic success rate. Similarly, operators with prior experience required 12 procedures, whereas those without required 27 to reach an acceptable false-negative rate (Figure 3). In the RA-CUSUM analysis, operators with prior experience required 11 procedures, while those without required 31 to achieve an acceptable diagnostic success rate. Likewise, operators with prior experience required 15 procedures, whereas those without required 42 to reach an acceptable false-negative rate.

**Figure 4:**
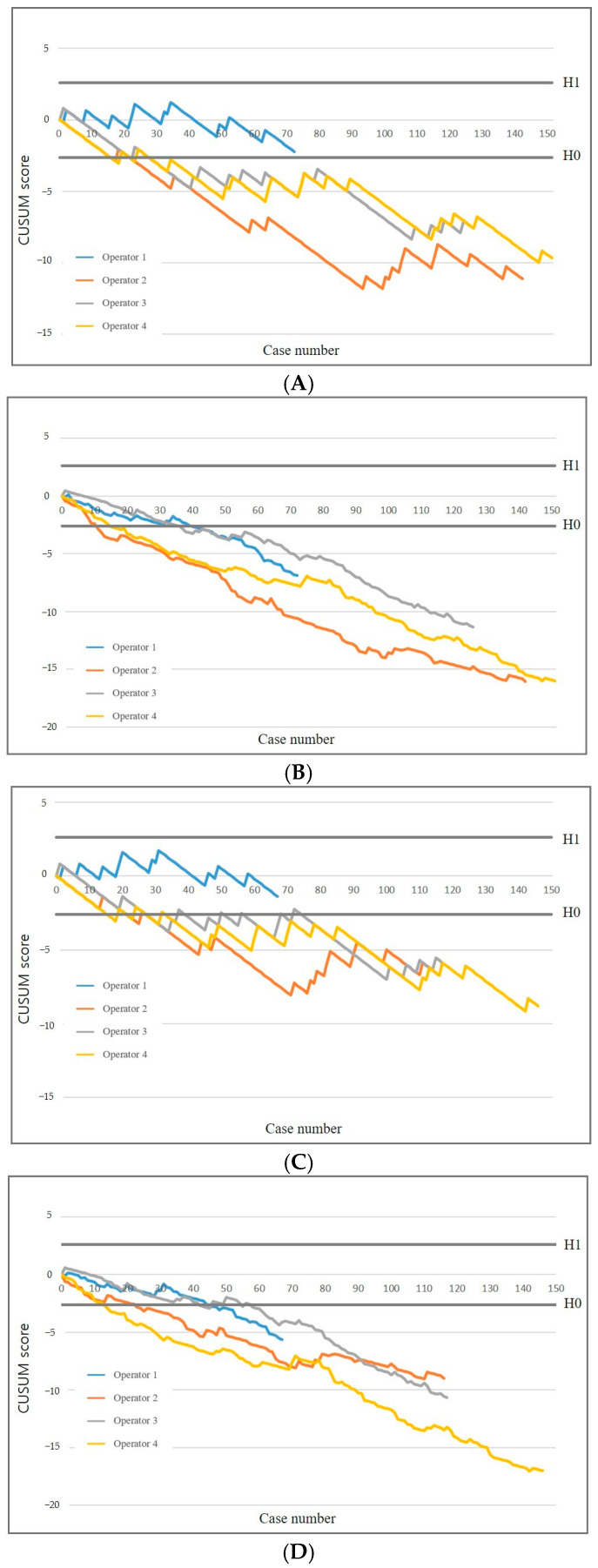
Effect of increasingly stringent failure criteria on CUSUM graphs. Standard (**A**) and risk-adjusted (**B**) CUSUM graphs of diagnostic success. Standard (**C**) and risk-adjusted (**D**) CUSUM graphs of false negative rate.

With a more stringent failure rate (p_0_ = 0.12, p_1_ = 0.24), three of the four operators attained an acceptable failure rate of 12% after a median of 22 PTNB procedures (range, 16–22) and an acceptable false-negative rate after a median of 22 procedures (range, 16–27) (Figure 4). Operator 1 did not achieve H0 and remained within the range between the decision thresholds. The RA-CUSUM chart demonstrated that all operators achieved both an acceptable failure rate and an acceptable false-negative rate. Although one operator did not meet the stricter threshold, the overall learning patterns remained consistent with those observed under the standard criterion (p_0_ = 0.16), supporting the robustness of operator performance across different thresholds.

### 3.3. Complication Occurrence

During US-guided PCPNB, a total of seven complications were observed, including one major complication. The major complication was a case of pneumothorax that required chest tube insertion for management. Among the minor complications, four cases of small pneumothorax and two cases of hematoma were identified. All minor complications were successfully treated with conservative management, without the need for further intervention. The occurrence of complications during the first and last 25 procedures performed by each operator was assessed. Among the first 25 procedures per operator, a total of four complications occurred. In contrast, no complications were observed during the last 25 procedures per operator (Table 2).

## 4. Discussion

US-guided PCPNB is a widely utilized technique for diagnosing indeterminate pleural lesions; however, its learning curve has not been well characterized [7,9,20]. Increased operator experience was associated with reduced rates of diagnostic failure, false negatives, and complications. Operators achieved an acceptable diagnostic success rate after a median of 15 procedures (range, 12–45) and an acceptable false-negative rate after a median of 18 procedures (range, 14–42) in the standard CUSUM analysis. In the RA-CUSUM analysis, operators achieved an acceptable diagnostic success rate after a median of 22 procedures (range, 10–33) and an acceptable false-negative rate after a median of 24 procedures (range, 12–44).

The diagnostic performance of US-guided PCPNB was 89.2%, comparable to prior studies (80–89%), despite the fact that our study did not utilize additional guidance tools such as contrast-enhanced US or ultrasound elastography [6,21]. Our study is the first to assess the learning curve for US-guided PCPNB, showing an acceptable diagnostic success rate after a median of 15 (range, 12–45) procedures in CUSUM and a median of 22 (range, 10–33) procedures in RA-CUSUM analysis. False negatives were the primary cause of diagnostic failure, and minimizing them is clinically crucial. Therefore, we performed additional analysis excluding benign lesions, which demonstrated acceptable false-negative rates after a median of 18 (range, 14–42) and 24 (range, 12–44) procedures, respectively. In comparison to the previous US-guided PCNB study for lung lesions, which assessed learning curves after at least 10 procedures, our study suggests that achieving proficiency in US-guided PCPNB requires more procedures [14]. This difference may be attributed to the challenges of pleural biopsy, including the variability in pleural morphology and pleural thickness, as well as the stricter acceptable failure rate in our study.

After RA-CUSUM adjustment for pleural morphology and thickness, the number of procedures required to reach proficiency decreased from a range of 12–45 (standard CUSUM) to 10–33, indicating a reduction in variability when accounting for case complexity. The amount of experience required to achieve proficiency may vary among operators and depends on the level of performance required [22]. Our study demonstrated considerable variability in the learning process for US-guided PCPNB. Such variability in the learning curve is consistent with findings from multiple studies across various procedural disciplines [10,11,23,24]. This variability may be partly explained by case complexity, including patient- or lesion-related factors that influence procedural difficulty. While RA-CUSUM modeling accounted for pleural morphology and thickness, other unmeasured factors—such as narrow intercostal spaces, limited cooperation, or lesion obscuration by bony structures—may also have contributed. These challenges are not systematically measurable in retrospective analyses but may affect operator performance and merit further prospective evaluation. Therefore, personalized training with expert guidance may be required.

A trend was observed where operators with prior PCNB experience reached the learning curve threshold faster than those without such experience. Although these operators had not previously performed biopsies on pleural lesions, their experience with PCNB in other organs likely refined their expertise in handling cutting needles and utilizing US guidance. Since pleural lesions, particularly those targeting the parietal pleura, are less affected by respiratory motion [25], the technical skills gained from PCNB in other organs may have been more easily adaptable to pleural biopsies.

Previous studies on US-guided biopsies of other organs have demonstrated that operator experience is closely associated with procedural performance [13,15,26]. For instance, in renal biopsies, experienced radiologists achieved higher sample adequacy than trainees, and in pediatric liver biopsies, operators with fewer than 10 cases had significantly higher rates of minor bleeding complications [13,15]. Additionally, in adult liver biopsies, experienced operators obtained longer tissue cores and more portal tracts, indicating higher specimen quality [26]. These findings suggest that, although US-guided biopsies involve similar core techniques, achieving procedural proficiency depends on adapting to the anatomical complexity of each target organ. In contrast, a study on US-guided PCPNB revealed variable CUSUM curves among radiologists performing the same procedure, indicating variability in individual learning trajectories. Accordingly, the learning curve for US-guided PCPNB should be interpreted in light of both organ-specific challenges and inter-operator variability in skill acquisition.

Our investigation demonstrated a minimal incidence of complications with US-guided PCPNB (six minor complications and one major complication), concordant with published meta-analytic data reporting aggregate complication rates of 3% (95% CI: 2–4%) and major adverse events at 1% (95% CI: 0–1%) [6]. The literature has established that post-procedural complications occur less frequently with US-guided versus CT-guided PCPNB—an observation further substantiated by our findings, highlighting the enhanced safety profile of US guidance for pleural biopsy procedures [9,27,28].

In this study, the threshold values used for CUSUM analysis (p_0_ = 0.16 and p_1_ = 0.32) were based on a recent meta-analysis reporting a pooled diagnostic yield of 0.84 for US-guided PCPNB [6,21]. These values were selected to reflect the expected performance of skilled operators under real-world conditions and to account for the inherent diagnostic uncertainty of the procedure. Notably, the pooled diagnostic yield from retrospective studies in the same meta-analysis was slightly lower, at 0.81, suggesting that our chosen p_0_ value of 0.16 may in fact represent a stricter standard than what is typically observed in routine clinical practice [6]. To complement the primary analysis, we additionally evaluated the learning curve using a more conservative threshold (p_0_ = 0.12). This analysis provided comparable learning patterns, suggesting that the observed trends were not substantially altered under stricter performance criteria. Although one operator did not meet the predefined proficiency level under this stricter threshold, the overall learning trends remained consistent, supporting the robustness of operator performance across varying diagnostic criteria.

Our study has several limitations. First, the study is retrospective; unrecorded factors during the procedures that could have influenced the diagnostic performance remain unknown. In particular, unmeasured variables such as poor acoustic windows, challenging lesion locations, or limited patient cooperation may have contributed to variability in procedural difficulty but could not be systematically assessed. In addition, the exclusion of indeterminate cases may have introduced selection bias, although their small number and balanced distribution likely limited the impact. Second, setting different failure rate thresholds may lead to substantially different outcomes, regardless of operator proficiency, necessitating careful interpretation of the CUSUM charts. Third, the limited number of operators in our study may affect generalizability; however, our diagnostic performance and complication rates align with the published literature, suggesting the reliability of our data. Further studies with more operators are needed to establish the precise number of procedures required to achieve proficiency. While operators were grouped by prior biopsy experience, further studies are needed to assess whether the specific organ biopsied affects the pleural biopsy learning curve. Lastly, prospective validation is needed to control for unmeasured variables such as lesion location and patient-related factors.

No guidelines have been published on how to assess operator’s performance in US-guided PCPNB procedures. This study provides valuable insights into the learning curve for US-guided PCPNB, demonstrating that operators achieve acceptable diagnostic success and false-negative rates with increasing procedural experience. The findings support the establishment of standardized training protocols and competency benchmarks for this procedure, ultimately enhancing procedural safety and diagnostic reliability in clinical practice.

## Figures and Tables

**Table 1:** Patient (*n* = 465) and lesion (*n* = 491) characteristics.

	Value
Sex	
Female	165 (35.5)
Male	300 (64.5)
Age (y), mean ± SD (median)	70.3 ± 12.1 (72)
Pleural thickness (mm), mean ± SD (median)	9.6 ± 6.6 (7.9)
Cases stratified by pleural thickness	
<4.5 mm	94 (19.1)
≥4.5 mm	397 (80.9)
Cases stratified by nodular morphology	
Nodular type	343 (69.9)
Diffuse type	148 (30.1)
No. of cores obtained, mean ± SD (range)	2.40 ± 0.92 (1–6)
Cases stratified by no. of cores obtained	
One or two cores	293 (59.7)
Three or more cores	198 (40.3)
Diagnosis of malignant lesions (*n* = 446)	
Lung cancer	372 (83.4)
Metastasis	38 (8.5)
Thymic epithelial tumor	11 (2.5)
Mesothelioma	11 (2.5)
Lymphoma	8 (1.8)
Sarcoma	5 (1.1)
Leukemia	1 (0.2)
Diagnosis of benign lesions (*n* = 45)	
Tuberculosis	24 (53.3)
Nonspecific benign	16 (35.6)
Solitary fibrous tumor	4 (8.9)
Fungal infection	1 (2.2)

Note—Unless otherwise indicated, data represent the number of patients or lesions, with percentages in parentheses.

**Table 2:** Operator Performance in Terms of Diagnostic Success and Complications.

	Operator 1	Operator 2	Operator 3	Operator 4
Number of PCPNBs	72	142	126	151
Diagnostic success †				
Overall	62 (86.1)	129 (90.8)	112 (88.9)	135 (89.4)
Initial 25	20 (80.0)	23 (92.0)	21 (84.0)	22 (88.0)
Final 25	22 (88.0)	24 (96.0)	23 (92.0)	24 (96.0)
Complication occurrence *				
Overall	1 (1.4)	2 (1.4)	1 (0.8)	3 (2.0)
Initial 25	1 (1.4)	1 (0.7)	1 (0.8)	1 (0.7)
Final 25	0	0	0	0

Data are numbers of patients, and data in parentheses are percentages. † Diagnostic success counts: overall, initial 25 cases, and final 25 cases; * complication occurrence counts: overall, initial 25 cases, and final 25 cases. PCPNB = percutaneous pleural needle biopsy.

**Table 3:** Overview of analysis results across different endpoints.

Outcome	Failure Rate Thresholds		Procedures Required to Reach Target by Operator			
	Acceptable	Unacceptable	Operator 1	Operator 2	Operator 3	Operator 4
Proposed failure rates						
Diagnostic success						
Standard	0.16	0.32	45	12	17	12
Risk-adjusted	0.16	0.32	30	10	33	14
False-negative rate						
Standard	0.16	0.32	42	17	19	14
Risk-adjusted	0.16	0.32	44	13	34	12
More stringent failure rate						
Diagnostic success						
Standard	0.12	0.24	Not Achieved	16	22	16
Risk-adjusted	0.12	0.24	40	11	36	15
False-negative rate						
Standard	0.12	0.24	Not Achieved	22	27	16
Risk-adjusted	0.12	0.24	45	23	42	13
Complication occurrence rate	0.02	0.06	Not Achieved	99	74	124

## Data Availability

The datasets presented in this article are not readily available because data sharing is restricted by the conditions of the Institutional Review Board (IRB) approval.

## Supplementary Materials

The following supporting information can be downloaded at https://www.mdpi.com/article/10.3390/diagnostics15131613/s1, Table S1: Prior Experience with Ultrasound-guided Percutaneous Needle Biopsies During Residency.

## References

[1] Bødtger U., Halifax R.J. (2020). Epidemiology: Why is pleural disease becoming more common?. Pleural Disease.

[2] Leung A.N., Müller N., Miller R.R. (1990). CT in differential diagnosis of diffuse pleural disease. AJR Am. J. Roentgenol..

[3] Jany B., Welte T. (2019). Pleural effusion in adults—Etiology, diagnosis, and treatment. Dtsch. Ärzteblatt Int..

[4] Lee P., Hsu A., Lo C., Colt H.G. (2007). Prospective evaluation of flex-rigid pleuroscopy for indeterminate pleural effusion: Accuracy, safety and outcome. Respirology.

[5] Boy D., Shaw J.A., Koegelenberg C.F.N. (2023). Ultrasound-guided pleural biopsy. Eurasian J. Pulmonol..

[6] Mei F., Bonifazi M., Rota M., Cirilli L., Grilli M., Duranti C., Zuccatosta L., Bedawi E.O., McCracken D., Gasparini S. (2021). Diagnostic yield and safety of image-guided pleural biopsy: A systematic review and meta-analysis. Respiration.

[7] Park J., Park B., Lim J.-K., Shin K.M., Lee J., Kim C.H., Seo H., Lee Y.H., Heo J., Do Y.W. (2021). Ultrasound-guided percutaneous needle biopsy for small pleural lesions: Diagnostic yield and impact of CT and ultrasound characteristics. Am. J. Roentgenol..

[8] Corcoran J.P., Tazi-Mezalek R., Maldonado F., Yarmus L.B., Annema J.T., Koegelenberg C.F., St Noble V., Rahman N.M. (2017). State of the art thoracic ultrasound: Intervention and therapeutics. Thorax.

[9] Sconfienza L.M., Mauri G., Grossi F., Truini M., Serafini G., Sardanelli F., Murolo C. (2013). Pleural and peripheral lung lesions: Comparison of US-and CT-guided biopsy. Radiology.

[10] Ahn S.Y., Park C.M., Yoon S.H., Kim H., Goo J.M. (2019). Learning curve of C-arm cone-beam computed tomography virtual navigation-guided percutaneous transthoracic needle biopsy. Korean J. Radiol..

[11] Park R., Lee S.M., Kim S., Park S., Choe J., Do K.-H., Seo J.B. (2022). Learning curve for CT-guided percutaneous transthoracic needle biopsy: Retrospective evaluation among 17 thoracic imaging fellows at a tertiary referral hospital. Am. J. Roentgenol..

[12] Page E.S. (1954). Continuous inspection schemes. Biometrika.

[13] Westheim B.H., Aagenæs I., Østensen A.B., Sanengen T., Almaas R. (2013). Effect of operator experience and frequency of procedure performance on complication rate after ultrasound-guided percutaneous liver biopsies. J. Pediatr. Gastroenterol. Nutr..

[14] Laursen C., Naur T.M.H., Bodtger U., Konge L., Henriksen D.P., Colella S., Naqibullah M., Minddal V., Davidsen J.R., Hansen N.C. (2016). Learning Curves for Ultrasound Guided Lung Biopsy in the Hands of Respiratory Physicians.

[15] Ferguson C., Winters S., Jackson S., McToal M., Low G. (2018). A retrospective analysis of complication and adequacy rates of ultrasound-guided native and transplant non-focal renal biopsies. Abdom. Radiol..

[16] Sacks D. (2003). Society of interventional radology clinical practice guidelines. J. Vasc. Interv. Radiol..

[17] Bolsin S., Colson M. (2000). The use of the Cusum technique in the assessment of trainee competence in new procedures. Int. J. Qual. Health Care.

[18] Sheth R.A., Baerlocher M.O., Connolly B.L., Dariushnia S.R., Shyn P.B., Vatsky S., Tam A.L., Gupta S. (2020). Society of interventional radiology quality improvement standards on percutaneous needle biopsy in adult and pediatric patients. J. Vasc. Interv. Radiol..

[19] Wang J., Zhou X., Xie X., Tang Q., Shen P., Zeng Y. (2016). Combined ultrasound-guided cutting-needle biopsy and standard pleural biopsy for diagnosis of malignant pleural effusions. BMC Pulm. Med..

[20] Hallifax R.J., Corcoran J.P., Ahmed A., Nagendran M., Rostom H., Hassan N., Maruthappu M., Psallidas I., Manuel A., Gleeson F.V. (2014). Physician-based ultrasound-guided biopsy for diagnosing pleural disease. Chest.

[21] Zhang Q., Deng M.-M., Li X.-L., Lu Y., Hou G. (2023). Thoracic ultrasound-guided real-time pleural biopsy in the diagnosis of pleural diseases: A systematic review and meta-analysis. Expert Rev. Respir. Med..

[22] Dessolle L., Fréour T., Barrière P., Jean M., Ravel C., Daraï E., Biau D.J. (2010). How soon can I be proficient in embryo transfer? Lessons from the cumulative summation test for learning curve (LC-CUSUM). Hum. Reprod..

[23] Komatsu R., Kasuya Y., Yogo H., Sessler D.I., Mascha E., Yang D., Ozaki M. (2010). Learning curves for bag-and-mask ventilation and orotracheal intubation: An application of the cumulative sum method. Anesthesiology.

[24] Kemp S., El Batrawy S., Harrison R., Skwarski K., Munavvar M., Roselli A., Cusworth K., Shah P. (2010). Learning Curves for Endobronchial Ultrasound Using Cusum Analysis.

[25] Kim J.H., Butler J.P., Loring S.H. (2011). Influence of the softness of the parietal pleura on respiratory sliding mechanisms. Respir. Physiol. Neurobiol..

[26] Chevallier P., Ruitort F., Denys A., Staccini P., Saint-Paul M.C., Ouzan D., Motamedi J.P., Tran A., Schnyder P., Bruneton J.N. (2004). Influence of operator experience on performance of ultrasound-guided percutaneous liver biopsy. Eur. Radiol..

[27] Yamamoto N., Watanabe T., Yamada K., Nakai T., Suzumura T., Sakagami K., Yoshimoto N., Sato K., Tanaka H., Mitsuoka S. (2019). Efficacy and safety of ultrasound (US) guided percutaneous needle biopsy for peripheral lung or pleural lesion: Comparison with computed tomography (CT) guided needle biopsy. J. Thorac. Dis..

[28] Khosla R., McLean A.W., Smith J.A. (2016). Ultrasound-guided versus computed tomography-scan guided biopsy of pleural-based lung lesions. Lung India.

