# Supplementary material for: Learning Curve of Ultrasound-Guided Percutaneous Needle Biopsy for Pleural Lesions: A Retrospective Study at Two Tertiary Referral Hospitals
Source: Diagnostics (Basel). 2025 Jun 25;15(13):1613. doi: 10.3390/diagnostics15131613 (PMC12249106; doi:10.3390/diagnostics15131613)
Supplement: Supplementary file 1 [file diagnostics-15-01613-s001.zip › diagnostics-3601061-supplementary.pdf]

Supplementary Table 1. Prior Experience with Ultrasound-guided Percutaneous Needle Biopsies During Residency

Operator	Biopsy site			Total
	Lung	Liver	Kidney	
1	0	0	0	0
2	23	18	2	43
3	0	0	0	0
4	27	16	3	46
